# Transmembrane Protein Ttyh1 Maintains the Quiescence of Neural Stem Cells Through Ca^2+^/NFATc3 Signaling

**DOI:** 10.3389/fcell.2021.779373

**Published:** 2021-11-16

**Authors:** Yuan Cao, Hai-ning Wu, Xiu-li Cao, Kang-yi Yue, Wen-juan Han, Zi-peng Cao, Yu-fei Zhang, Xiang-yu Gao, Ceng Luo, Xiao-fan Jiang, Hua Han, Min-hua Zheng

**Affiliations:** ^1^ Department of Neurosurgery, Xijing Hospital, Fourth Military Medical University, Xi’an, China; ^2^ State Key Laboratory of Cancer Biology, Department of Biochemistry and Molecular Biology, Fourth Military Medical University, Xi’an, China; ^3^ Department of Medical Genetics and Developmental Biology, Fourth Military Medical University, Xi’an, China; ^4^ Department of Neurobiology, Fourth Military Medical University, Xi’an, China; ^5^ Department of Occupational and Environmental Health, Fourth Military Medical University, Xi’an, China

**Keywords:** neural stem cell, Ttyh1, quiescence, neurogenesis, stemness

## Abstract

The quiescence, activation, and subsequent neurogenesis of neural stem cells (NSCs) play essential roles in the physiological homeostasis and pathological repair of the central nervous system. Previous studies indicate that transmembrane protein Ttyh1 is required for the stemness of NSCs, whereas the exact functions *in vivo* and precise mechanisms are still waiting to be elucidated. By constructing Ttyh1-promoter driven reporter mice, we determined the specific expression of Ttyh1 in quiescent NSCs and niche astrocytes. Further evaluations on Ttyh1 knockout mice revealed that Ttyh1 ablation leads to activated neurogenesis and enhanced spatial learning and memory in adult mice (6–8 weeks). Correspondingly, Ttyh1 deficiency results in accelerated exhaustion of NSC pool and impaired neurogenesis in aged mice (12 months). By RNA-sequencing, bioinformatics and molecular biological analysis, we found that Ttyh1 is involved in the regulation of calcium signaling in NSCs, and transcription factor NFATc3 is a critical effector in quiescence versus cell cycle entry regulated by Ttyh1. Our research uncovered new endogenous mechanisms that regulate quiescence versus activation of NSCs, therefore provide novel targets for the intervention to activate quiescent NSCs to participate in injury repair during pathology and aging.

## Highlights

1) Ttyh1 is expressed in Ki67-negative quiescent neural stem cells.
2) Ttyh1 ablation leads to increased neurogenesis and enhanced spatial learning and memory in adult mice.
3) Ttyh1 deficiency results in accelerated exhaustion of neural stem cell pool in aged mice.
4) Ttyh1 hinders the transition from quiescence to activation in NSCs by activating Ca^2+^/NFAT signaling.

## Introduction

Neural stem cells (NSCs) in the neurogenic niches of the adult mammalian brain are in different quiescent states, proliferate and generate descendant cells, which contribute to the preexisting neuronal networks and affect animal behaviors (Codega et al., 2014; Urbán et al., 2019). The state of quiescence protects the long-lived adult NSCs from early exhaustion and replicative damage that might lead to malignant transformation or senescence (Cheung and Rando 2013). Therefore, quiescent NSCs (qNSCs) provide important source of the lifelong adult neurogenesis, which holds great significance in maintaining brain tissue homeostasis and functional plasticity. However, the biological mechanisms of quiescence and timely activation are still unclear.

**GRAPHICAL ABSTRACT:**
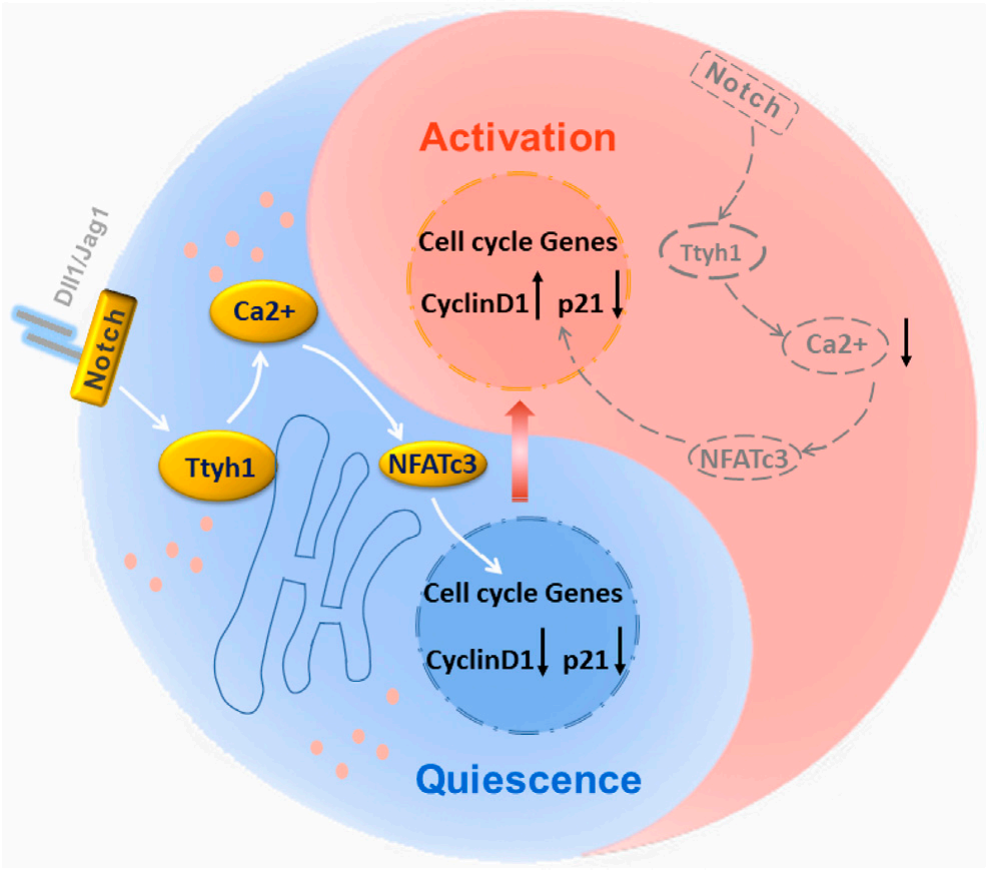


There are two main neurogenic niches in the adult mammalian brain, the subventricular zone (SVZ) adjacent to the ependyma and the subgranular zone (SGZ) of the hippocampal dentate gyrus. The identities of NSC subgroups in neurogenic niches are dynamic, with different metabolic characteristics, diverse physiological activities, and dissimilar molecular markers (Ming and Song 2011; Bond et al., 2015; Zywitza et al., 2018). For instance, rodent NSCs in SVZ contain GFAP^+^Sox2^+^ type-B NSCs, which activate and express Ki67 and MCM2, give rise to EGFR^+^ transient amplifying progenitors (TAPs, type-C cells) and then differentiate into DCX^+^ neuroblasts (type-A cells), and finally generate newborn interneurons to supply olfactory neuronal pool via rostral migratory stream (RMS). It is important to find subgroup-specific molecules, which might regulate the transition between different development stages, especially from quiescence to activation.

The *Tweety (tty)* gene was originally identified as a transcription unit by Campbell in a region of the *flightless (fli)* locus of *Drosophila melanogaster* (Campbell et al., 1993). In vertebrate, the *Tweety* family contains three genes, Ttyh1, Ttyh2 and Ttyh3, and the proteins encoded by them contain five transmembrane regions in similar arrangement (Halleran et al., 2015; Han et al., 2019). Ttyh1 contains 450 amino acid residues, and mainly locates in the endoplasmic reticulum (ER) and Golgi apparatus of specific cells in the brain, eyes, spinal cord, and testis (Kumada et al., 2010; Wiernasz et al., 2014). Previous studies suggested that Ttyh1 mainly functions as a volume-regulated chloride channel (Suzuki 2006; Bae et al., 2019; Han et al., 2019). In the brain, Ttyh1 is highly expressed in neural stem/precursor cells (Halleran et al., 2015; Okamoto et al., 2016; Cao et al., 2017), and is required for the maintenance of NSC properties (Kim et al., 2018). Further *in vivo* analysis by our research group showed that knockout of Ttyh1 leads to enhanced neurogenesis, suggesting that it may be an important regulator on quiescence/activation switch for NSCs (Wu et al., 2019). In the mature neuron compartment, we have also shown that Ttyh1 participates pain perception (Han et al., 2021). However, despite these findings, the precise role of Ttyh1 in NSCs *in vivo* and its specific biological mechanisms still await elucidation.

Here we further investigated the expression and distribution of Ttyh1 in NSCs, TAPs and neuroblasts by immunofluorescence and Ttyh1 promoter-driven reporter mice, and determined the biological roles of Ttyh1 by *in vivo* knockout and *in vitro* knockdown models. We further clarified the specific molecular mechanism of Ttyh1 on the regulation of NSCs by RNA-sequencing. These studies uncovered that Ttyh1 is specifically expressed in Ki67^-^ qNSCs, and plays essential roles in the quiescence/activation transition, stemness maintenance, and neurogenesis of adult NSCs in mice. Our research revealed new mechanisms that regulate quiescent state and timely activation of NSCs, and might provide a novel target and intervention strategy for activating qNSCs participating in injury repair and aging.

## Materials and Methods

### Animals

Ttyh1 knockout mice were constructed with exon 4 of Ttyh1 deleted, which result in a truncated protein of 198 amino acids (140 native amino acids plus 58 frame-shift amino acids), thus disrupts the Ttyh1 gene (Wu et al., 2019). Ttyh1-CreERT2 mice were constructed by using the CRISPR/Cas9 system to knock into P2A-iCreERT2 at exon 13 of Ttyh1 gene, with its ORF intact. Ttyh1 reporter mice were generated by crossing Ttyh1-CreERT2 mice that harbored a tamoxifen-inducible CreERT2 fusion gene with Ai9 flox-stop-flox-mT mice harboring loxP sites on either side of a STOP sequence, upstream of a membrane-targeted tdTomato (mT) cassette, referred to as Ttyh1-CreERT2; Rosa-tdTomato mice.

All animal procedures used in this study were performed in accordance with protocols approved by the Animal Experiment Administration Committee of the Fourth Military Medical University to ensure ethnical and humane treatment of animals. All transgenic mice used in this study were backcrossed for at least six generations and maintained on a C57BL/6 background. Animals were housed in a 12 h light/12 h dark cycle with food and water ad libitum.

### Tamoxifen Administration

Tamoxifen (Sigma-Aldrich, St. Louis, MO, United States) was dissolved in corn oil with the final concentration of 20 mg/ml, and was confirmed to be completely dissolved by vortex oscillation before injections. Then Tamoxifen was intraperitoneally injected with 75 mg/kg dosage for five consecutive days. In order to observe the *in situ* and early migrated Ttyh1 positive cells and progeny cells, we took samples on the 5th day and 10th day post induction (5 and 10 dpi, respectively).

### BrdU Incorporation

For 5-bromo-2-deoxyuridine (BrdU) labeling, BrdU (50 mg/kg, Sigma-Aldrich) was injected intraperitoneally into mice every 2 h for 5 times in 1 day. Brain tissues were taken 2 h after the last injection and immunostained with anti-BrdU (1:250, Abcam, Cambridge, United Kingdom) by immunofluorescence.

### Tissue Processing and Immunofluorescence Staining

After anesthesia, the mice were perfused with PBS, and the whole brains were removed, coated with OCT compound (Sigma-Aldrich), and stored at −80°C. These brain tissues were serially sectioned with 14 μm depth using a cryostat (Leica, CM 1950), and then stored at −20°C. Brain sections were fixed with 4% PFA for 5 min before staining, and then washed with PBS for 3 times. They were incubated in PBS/0.1% Triton X-100 containing 1% BSA for 30 min and then incubated with primary antibodies overnight at 4°C. The primary antibodies used for incubation are as follows: rabbit anti-Sox2 (1:400, Abcam), goat anti-GFAP (1:400, Abcam), rabbit anti-Doublecortin (DCX) (1:400, Abcam), rabbit anti-EGFR (1:400, Abcam), rabbit anti-γ-tubulin (1:400, Abcam), rat anti-CD24 (1:400, BD pharmingen, San Diego, CA, United States). Brain sections were washed in PBS for 3 times and then incubated with secondary antibodies for 2 h at room temperature. After 3 times of washes, sections were mounted with mounting media (Vector Labs, Burlingame, CA, United States) containing DAPI for nuclear staining. Brain tissues from Ttyh1-CreERT2; Rosa-tdTomato mice were fixed with 4% PFA after PBS perfusion, dehydrated with 30% sucrose solution for 48 h, and then frozen and sectioned. The stained brain sections were visualized and imaged by a fluorescence microscope (Eclipse Ni, Nikon) or a laser scanning confocal microscope (A1R confocal microscope, Nikon).

### Primary NSCs Culture and Lentivirus Infection

NSCs were derived from embryonic brain tissues of E14.5 embryos. Pregnant mice were anesthetized and embryos were dissected. After removing the brain meninges, the forebrain tissues of fetal mice were cut into pieces, and single cell suspensions were obtained by mechanical dissociation under a stereomicroscope. Then cells were cultured in Neurobasal medium (Thermo Fisher Scientific, Waltham, MA, United States) supplemented with N2 (Gibco, Grand Island, NY, United States), B27 (Gibco), 20 ng/ml recombinant human basic fibroblast growth factor (bFGF, R&D Systems, Minneapolis, MN, United States), 20 ng/ml recombinant human epidermal growth factor (EGF, R&D Systems), 1% Penicillin/Streptomycin (Gibco), L-Glutamine (100 X, Gibco) and heparin (1 U/mL, Sigma-Aldrich), and cultured in incubator at 37°C.

Lentiviruses were produced by Shanghai Genechem Co., Ltd. The short hairpin RNA (shRNA) sequences were as follows: Ttyh1-targeting shRNA (shTtyh1): 5′-CCG​GCG​GCA​TTG​GCA​TTG​GTT​TCT​ACT​CGA​GTA​GAA​ACC​AAT​GCC​AAT​GCC​GTT​TTT​G-3′. For lentiviral infection, NSCs were digested into single cells by accutase (Gibco) and plated into 6-well plates with 1 × 10^6^ cells in each well. According to the method of suspension transfection, fresh medium containing concentrated shTtyh1 lentivirus was added into the 6-well plate, with lentivirus contained empty vector as control. After 12 h of incubation, the medium was replaced by the ordinary NSC culture medium (as described above). At 48 h and 72 h after infection, cells were observed and photographed. And 72 h after infection, cells were collected for immunofluorescence staining, RT-qPCR or Western blotting analysis.

### Cellular Immunofluorescence Staining

Cells were plated on coverslips for 24 h incubation, and were fixed in 4% PFA for 30 min at room temperature. Samples were incubated with primary antibodies overnight at 4°C, followed by Alexa Fluor^®^ 488-conjugated or Alexa Fluor^®^ 594-conjugated second antibodies (1:500, Jackson Immunoresearch Laboratories, West Grove, PA, United States) for 2 h at room temperature. The primary antibodies used for incubation are as follows: rabbit anti-Sox2 (1:400, Abcam), goat anti-GFAP (1:400, Abcam), rabbit anti-Doublecortin (DCX) (1:400, Abcam), rabbit anti-EGFR (1:400, Abcam), rabbit anti-Map2 (1:800, Sigma-Aldrich), rabbit anti-Oligodendrocyte Marker O4 (1:100, Sigma-Aldrich). The stained sections were imaged by a fluorescence microscope (Eclipse Ti, Nikon).

### Open Field Test

The inner and bottom walls of the open field reaction box were disinfected with 75% alcohol in advance to avoid the interference of residual smell on the experimental results. During the open field test, the experimental environment was kept quiet. The general recording time was 5 min per mouse. And the movement speed, the total movement distance, the activity time in the central area, and the activity time in the peripheral area of mice were recorded.

### Morris Water Maze

For the first 5 days, the mice were pre-trained in the water maze. Each mouse was placed in the circular pool from 4 different quadrants in sequence, and was trained to find the platform, and the time the mice used to find the platform in the water was recorded. If the mouse did not find the platform within the limited time of 60 s, the mouse was artificially guided to the platform, and was allowed to stay on the platform for at least 10 s. On the 6th test day, the platform in the pool was removed, the mice in each group were placed in a circular pool from a random quadrant, and the activity time of the mice in the target quadrant was recorded.

### Intercellular Calcium Analysis

NSCs from E14.5 control and Ttyh1 knockout embryonic mice were digested into single cells, and then counted and seeded in 24-well plates at 5 × 10^5^ cells per well. 24 h later, cells were washed twice with ACSF, and the Fluo-8 dye-loading solution (Abcam) was added into the cell culture medium at the concentration of 4 μM. The cells were incubated for 1 h at 37°C and washed twice with ACSF. Run the calcium flux assay by monitoring the fluorescence intensity at Ex/Em = 490/525 nm. The ER calcium release and store-operated calcium entry (SOCE) were induced by adding 10 μM thapsigargin (Abcam) and 2 μM calcium chloride solution into the culture medium, respectively. The changes of intracellular calcium were detected. After acquisition, single cells were segmented manually and analyzed for Fluo-8 fluorescence using ImageJ.

### Single Cell RNA Sequencing Data Analysis

We analyzed the expression profile of single cell RNA sequence with accession number GEO: GSE147191 and BioProject: PRJNA324289. We employed a global-scaling normalization method “LogNormalize” that normalizes the gene expression measurements for each cell by the total expression, multiplies this by a scale factor (10,000 by default), and log-transforms the result. The formula is shown as follows: A gene expression level = log(1 + (UMI A ÷ UMI Total) × 10,000). Seurat implements a graph-based clustering approach. Distances between the cells were calculated based on previously identified PCs. We first constructed a KNN graph based on the Euclidean distance in PCA space, and refined the edge weights between any two cells based on the shared overlap in their local neighborhoods (Jaccard distance). To cluster the cells, we applied modularity optimization techniques SLM, to iteratively group cells together, with the goal of optimizing the standard modularity function. We used t-SNE to put cells with similar local neighborhoods in high-dimensional space together in low-dimensional space to visualize data information.

### RNA Sequencing and Data Analysis

After transfection with lentivirus containing shTtyh1 and negative control, the total RNA was extracted according to the manufacturer’s instructions and submitted to the Gene Denovo Biotechnology Co. (Guangzhou, China) for transcriptome sequencing using Illumina Novaseq6000. We carried out gene ontology (GO), Kyoto Encyclopedia of gene and genomes (KEGG), and gene set enrichment analysis (GSEA).

### Western Blotting

All cells were lysed with working solution of the RIPA buffer (Beyotime, Shanghai, China) containing a protease inhibitor cocktail (Roche, Basel, Switzerland). The protein concentration in the supernatants was determined by using the BCA Protein Assay kit (Thermo Fisher Scientific). The protein samples were separated by 12% SDS-PAGE and blotted onto polyvinylidene fluoride (PVDF) membranes (Merck Millipore, Billerica, MA, United States). Membranes were blocked using 5% skim milk at room temperature for 1 h, and incubated at 4°C overnight with the addition of the primary antibodies of β-actin (1:10,000, Abcam), Ttyh1 (1:400, self-prepared (Cao et al., 2017), STIM1 (1:400, Proteintech, Chicago, IL, United States), Orai1 (1:400, Proteintech), NFATc3 (1:500, Proteintech), CALM (1:1,000, Abcam), CaMKII (1:1,000, Abcam), Cyclin D1 (1:5,000, Proteintech), p21 (1:1,000, Abcam). After being rinsed with phosphate-buffered saline with Tween-20 (PBST) three times, the membrane was incubated with HRP-conjugated secondary antibody for 1h, followed by exposure and imaging using the enhanced chemiluminescence (ECL) detection kit (Merck Millipore).

### RT-qPCR Analysis

Total RNA was extracted using TRIzol reagent (Invitrogen, Carlsbad, CA, United States), Standard cDNA synthesis reactions were carried out using PrimeScript^®^ reverse transcriptase Master Mix (TaKaRa, Tokyo, Japan) reverse transcriptase according to the manufacturer’s instructions. For RT-qPCR analysis, reverse transcribed products were amplified using TB Green™ Premix Ex Taq™ II (TaKaRa). The following primers were used as followed:

**Table:** 

Gene name	Sense sequence/Antisense sequence
Ttyh1	Forward: 5′-CCG​CGA​CCA​AGA​GTA​CCA​G-3′
	Reverse: 5′-GAA​GCG​GAT​GAG​GTA​GAC​AGC-3′
STIM1	Forward: 5′-TGA​AGA​GTC​TAC​CGA​AGC​AGA-3′
	Reverse: 5′-AGG​TGC​TAT​GTT​TCA​CTG​TTG​G-3′
STIM2	Forward: 5′-CGA​AGT​GGA​CGA​GAG​TGA​TGA-3′
	Reverse: 5′-GGA​GTG​TTG​TTC​CCT​TCA​CAT​T-3‘
Orai1	Forward: 5’-GAT​CGG​CCA​GAG​TTA​CTC​CG-3′
	Reverse: 5′-TGG​GTA​GTC​ATG​GTC​TGT​GTC-3′
NFAT1	Forward: 5′-TCA​GAA​AGC​TGG​GTT​GGG​AC-3′
	Reverse: 5′-GCT​ATC​TGG​CTG​CAA​CTC​AAG-3′
NFAT2	Forward: 5′-CCC​TAT​CGA​GTG​TTC​CCA​GC-3′
	Reverse: 5′-TCA​AAG​TCG​TCC​GTG​GGT​TC-3′
NFATc3	Forward: 5′-GCT​CGA​CTT​CAA​ACT​CGT​CTT-3′
	Reverse: 5′-GAT​GTG​GTA​AGC​CAA​GGG​ATG-3′
NFATc4	Forward: 5′-GAT​CGA​GGT​ACA​GCC​TAG​AGC​A-3′
	Reverse: 5′-GCA​GAG​TCA​ATG​GCT​TCT​CAC​TG-3′
NFAT5	Forward: 5′-CAG​CGC​CCA​ATA​GTT​GGC​A-3′
	Reverse: 5′-TGC​TGG​TGA​AAA​ATT​GAC​TGG​T-3′
Ccnd1	Forward: 5′-GCA​GAA​GGA​GAT​TGT​GCC​ATC​C-3′
	Reverse: 5′-AGG​AAG​CGG​TCC​AGG​TAG​TTC​A-3′
p21	Forward: 5′-TCG​CTG​TCT​TGC​ACT​CTG​GTG​T-3′
	Reverse: 5′-CCA​ATC​TGC​GCT​TGG​AGT​GAT​AG-3′

### Quantification and Statistical Analysis

Images were analyzed using ImageJ software. All statistical tests and sample sizes (*n*) are included in the Figure Legends. All data are shown as mean ± SEM. All statistical analyses were performed using GraphPad Prism 8. Values of *p* ≤ 0.05 were considered statistically significant in the two-tailed Student’s *t* test, and not statistically significant (ns) when *p* > 0.05.

## Results

### Ttyh1 is Expressed in Quiescent NSCs in Neurogenic Niche

**FIGURE 1:**
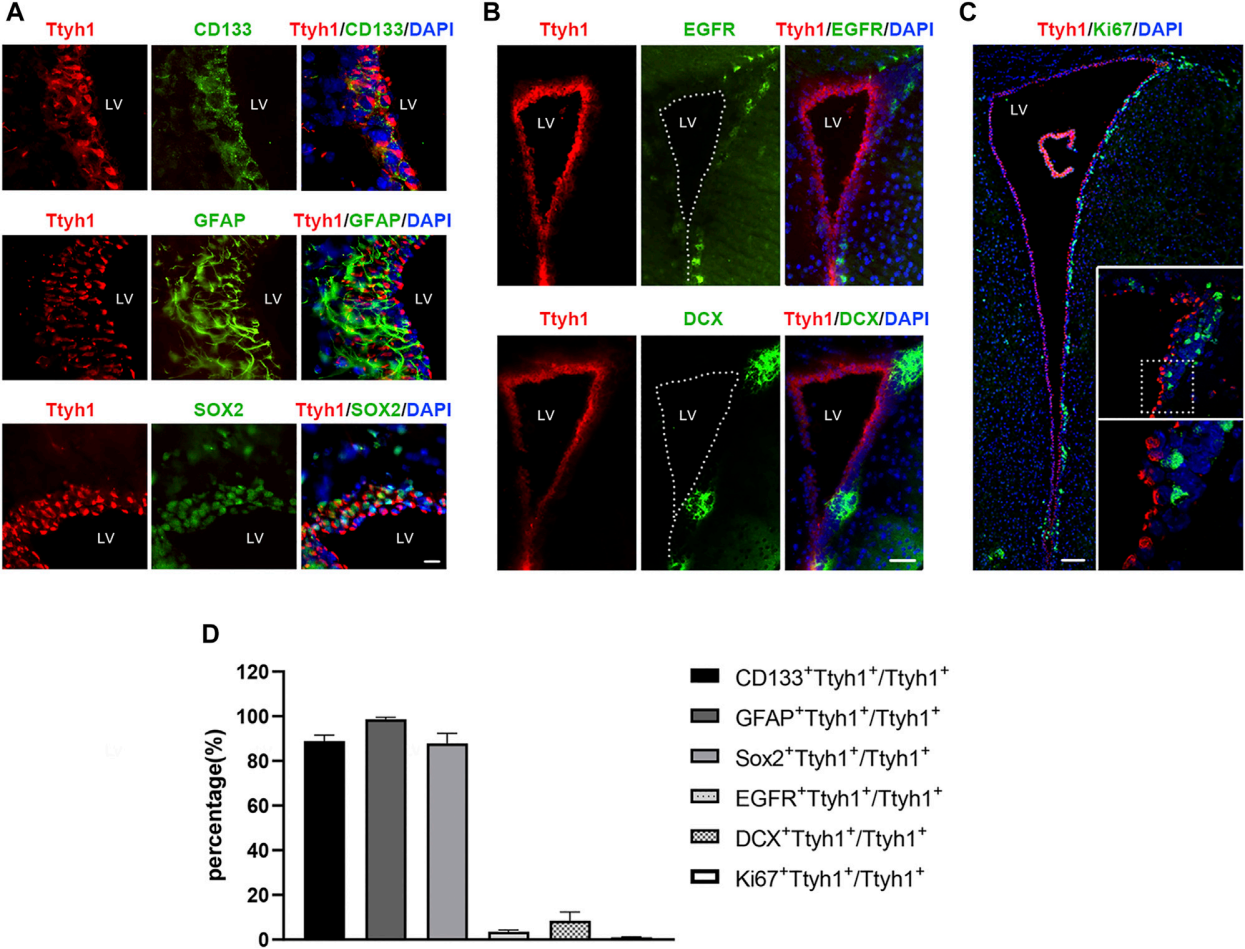
Ttyh1 is expressed in Ki67^-^ quiescent NSCs in SVZ. **(A)** Double-labeling of Ttyh1 and NSC markers. Ttyh1 co-labeled cells with CD133, GFAP and Sox2 at high frequency in SVZ. **(B)** Double-labeling of Ttyh1 and TAP or neuroblast cell markers. Ttyh1 seldomly labeled EGFR^+^ and DCX^+^ cells. **(C)** Double-labeling of Ttyh1 and proliferative cell marker Ki67. Ttyh1 completely were not co-labeled with Ki67 in entire SVZ region. The non-overlapping of Ttyh1^+^ and Ki67^+^ signals are showed in magnifications within white rectangles (the lower field is the magnification of the dotted rectangle in upper field). **(D)** Statistics of the proportions of co-labeled cells in Ttyh1^+^ cells. Scale bar = 10 μm in A, Scale bar = 50 μm in B, Scale bar = 100 μm in C, Scale bar = 10 μm in D. *n* = 3 for all experiments. Data are expressed as mean ± SEM. LV, lateral ventricle.

To study the expression pattern of Ttyh1 in NSCs and their descendant cells, we used a Ttyh1 antibody and cell-type marker antibodies to label Ttyh1^+^ cells in NSCs, TAPs and neuroblasts of 8 weeks old wild-type mice. We found that Ttyh1 was mostly co-labeled with NSC makers such as GFAP, CD133, and Sox2, with the proportions of double positive cells in total Ttyh1^+^ cells as 98.68 ± 0.69%, 88.94 ± 2.16%, and 87.90 ± 3.61%, respectively, (Figures 1A,D). On the other hand, Ttyh1 was seldom co-labeled with EGFR^+^ TAPs and DCX^+^ neuroblasts, with a few double positive cells in total Ttyh1^+^ cells (Figures 1B,D). Most importantly, Ttyh1 did not co-label with Ki67^+^ cells in entire SVZ region (Figures 1C,D), indicating that Ttyh1-labeled cells were in quiescence and did not proliferate. Quiescent NSCs have been previously identified to rest in G0 and G2 phases (Otsuki and Brand 2018). Therefore, these results suggest a major constituent of Ttyh1^+^ stem cells in the total NSC pool and especially the qNSC subgroup in the adult neurogenic niche.

**FIGURE 2:**
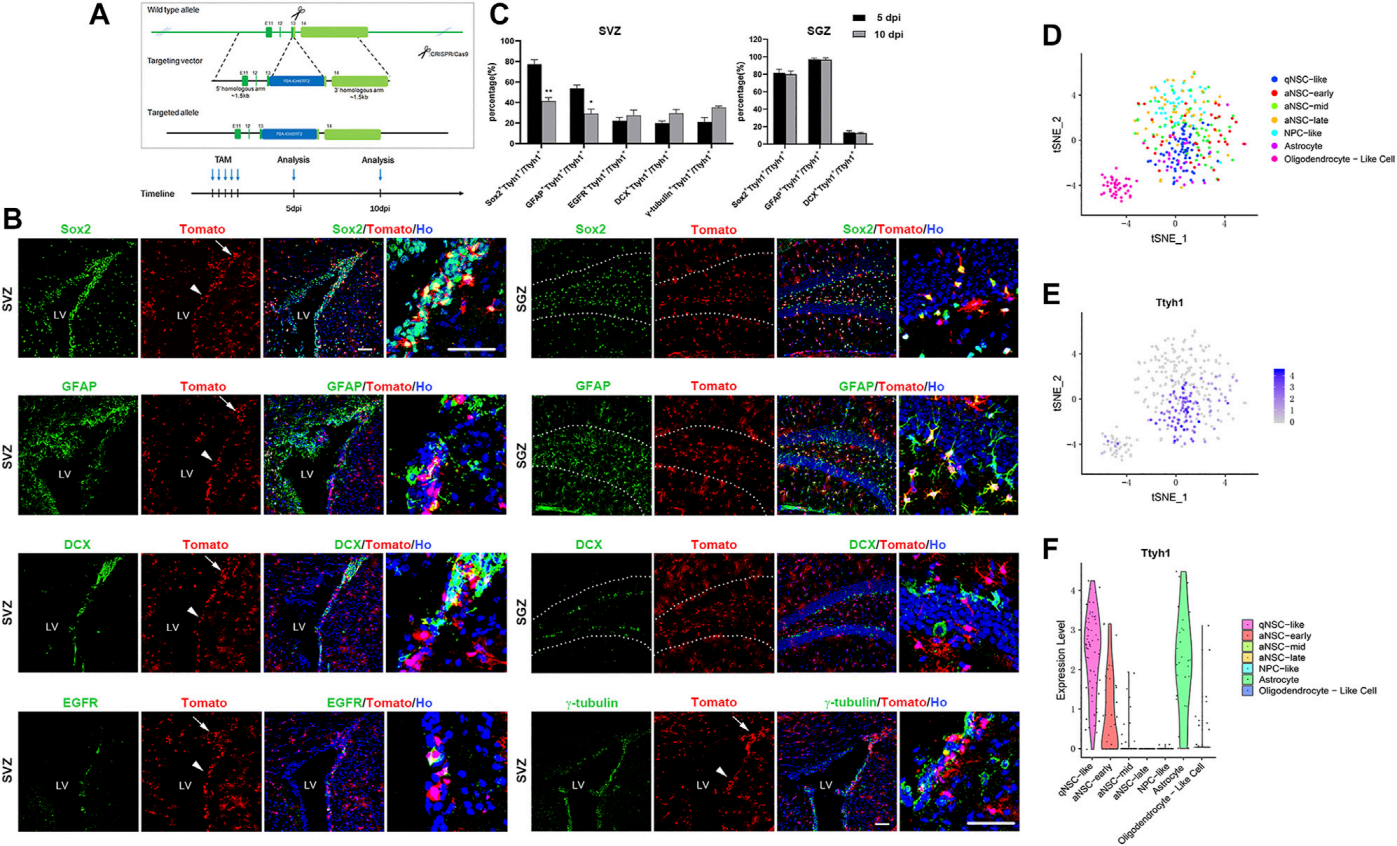
Ttyh1 is mainly distributed in qNSCs and astrocytes in neurogenic niche. **(A)** Diagrams of Ttyh1-CreERT2 mice construction and tamoxifen induction strategy. Targeting vector introduced P2A-iCreERT2 into the 13th exon located at the 3’ terminal of *Ttyh1* gene to construct Ttyh1-CreERT2 mice, with Ttyh1 ORF intact. After crossing with Ai9 Rosa26-tdTomato reporter mice, Ttyh1-CreERT2; Rosa-tdTomato mice were obtained and were injected tamoxifen for continuous 5 days at 8 weeks old. Samples were taken at 5 days post induction (dpi) and 10 dpi for analysis. **(B)** Immunofluorescence analysis of reporter mice in neurogenic niches. The samples taken from 5 dpi group showed that Ttyh1-tomato^+^ cells in SVZ were mainly concentrated on the striatal side (arrowheads) and dorsal wedge (arrows). These Ttyh1-tomato^+^ cells mainly co-labeled with Sox2 and GFAP, and less co-labeled with DCX in SVZ and SGZ. Ttyh1-tomato^+^ cells also co-labeled with EGFR and γ-tubulin at a small proportion in SVZ. **(C)** Statistics of the proportions of co-labeled cells in Ttyh1-tomato^+^ cells. The proportions of Sox2^+^Ttyh1^+^ cells and GFAP^+^Ttyh1^+^ cells were significantly reduced at 10 dpi compared with those at 5 dpi. **(D)** Single-cell cluster analysis of single-cell RNA sequencing data showed the classification of adult NSCs in SVZ and their progeny cells. **(E)** The parallel analysis of D showed the distribution of Ttyh1 transcripts in cell clusters. Purple dots represent Ttyh1^+^ cells. **(F)** Violin plot of Ttyh1 distribution in NSCs and progeny cells based on single-cell RNA sequencing data. Ttyh1 was highly expressed in qNSCs and astrocytes (in accordance with accumulated Tomato^+^ cells in SVZ, and scattered Tomato^+^ cells outside SVZ in B, respectively). Scale bar = 50 μm in B. *n* = 3 for all experiments. Data are expressed as mean ± SEM. Statistical significance was calculated using an unpaired, two-tailed Student’s *t*-test. (∗∗) *p* < 0.01, (∗) *p* < 0.05. LV, lateral ventricle.

To further estimate the distribution of Ttyh1 in neurogenic niches, and track the progeny cells of Ttyh1^+^ NSCs, we constructed Ttyh1-CreERT2 transgenic mice (Figure 2A), which were crossed with Ai9 Rosa26-tdTomato reporter mice to obtain double transgenic offspring contains Ttyh1-CreERT2; Rosa-tdTomato. This will allow the *Cre* gene to be expressed after tamoxifen injection, to initiate tdTomato expression, and label cells expressing Ttyh1 and trace their progeny. Five days after induction on 8 weeks old adult mice, the brain tissues of control Rosa-tdTomato mice did not show fluorescent signals (data not shown), but the brain tissues of Ttyh1-CreERT2; Rosa-tdTomato mice showed intensive red fluorescent signals (Figure 2B). Five (Figures 2B,C) and 10 days (Figure 2C) after induction, Ttyh1^+^ cells were largely located in the striatal side in SVZ (arrowheads in Figure 2B), especially in the dorsal wedge (arrows in Figure 2B). Importantly, Ttyh1^+^ signals were observed in the subgranular layer within the dentate gyrus (dotted lined area in Figure 2B) of hippocampus. A large proportion of these cells were co-labeled with Sox2 and GFAP, with the percentages of double positive cells in total Ttyh1^+^ cells as 77.33 ± 3.54% and 53.67 ± 2.76% in SVZ, and as 81.71 ± 5.68% and 97.01 ± 1.27% in SGZ, respectively (Figure 2C), at 5 days post injection (5 dpi). The cells co-labeled with Ttyh1 and DCX are mainly located in the dorsal wedge of SVZ (Figure 2B), indicating that Ttyh1^+^ positive cells underwent neurogenesis process. We compared the proportions of co-labeled cells between 5 and 10 dpi, and found that the proportions of Sox2^+^Ttyh1^+^ cells and GFAP^+^Ttyh1^+^ cells in the total number of Ttyh1^+^ cells decreased significantly (*P* = 0.0029, *P* = 0.0119). The proportions of other types of cells increased slightly, although the results were not significant. This change showed the dynamic differentiation of NSCs labeled with Ttyh1. These results indicate that, on 10 dpi, some of the B-type cells labeled with Ttyh1 differentiated into progeny cells. Indeed, Ttyh1-tdTomato and EGFR, DCX co-label in the SVZ zone respectively, but with low percentages (Figure 2C), indicating that Ttyh1^+^ NSCs differentiate at a quite low frequency. In addition, the Ttyh1 signal in γ-tubulin^+^ ependymal cells was also limited (Figures 2B,C).

To further refine Ttyh1 expression in NSC subpopulations, we used the published single-cell sequencing data (Dulken et al., 2017; Donnard et al., 2020) to analyze Ttyh1 expression in various NSC subtypes by unsupervised clustering analysis (Figures 2D,E). The results showed that Ttyh1 was mainly expressed in qNSCs (Figure 2E). It began to decrease in the early activated NSCs (aNSCs), and gradually disappeared in the middle and late aNSCs. Violin plot clearly showed the process of Ttyh1 decreasing with the differentiation of NSCs (Figure 2F). In addition, Ttyh1 was highly expressed in niche astrocytes (Figure 2F).

Notably, we also observed Ttyh1^+^ cells outside the SVZ and SGZ region (Figure 2B). Some of these cells co-labeled with Sox2 and GFAP, suggesting they might be mature astrocytes. Further analysis on another single-cell sequencing data from mature neural cells confirmed this suggestion (Supplementary Figure S1).

### Ttyh1 Maintains NSC Stemness *in vitro*

**FIGURE 3:**
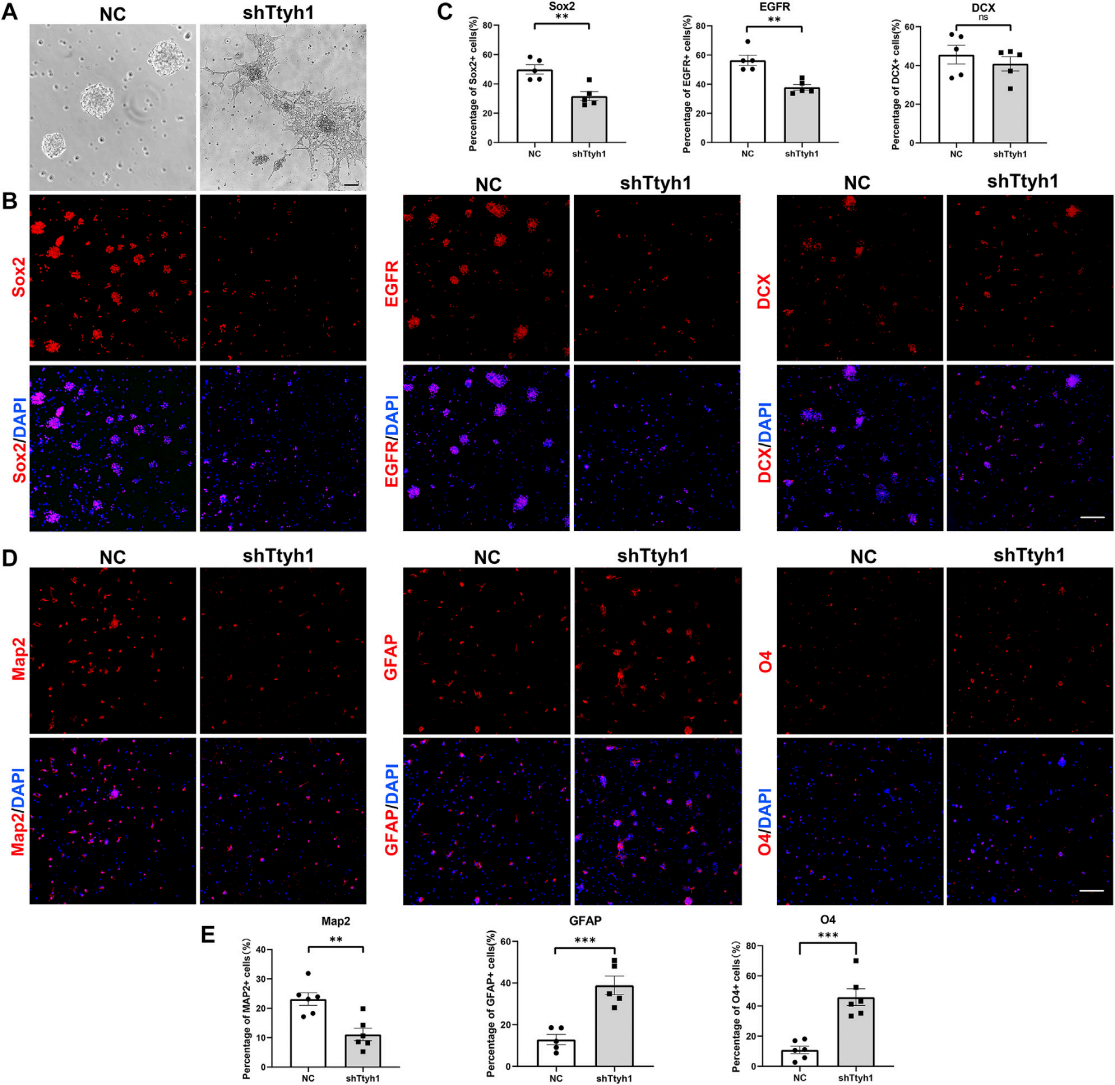
Ttyh1 maintains the stemness of NSCs *in vitro*. **(A)** Bright field microscopy showed the morphological disparity between NSCs transfected with negative control shRNA (NC) and shTtyh1. We added NC and shTtyh1 containing lentiviruses respectively on primary cultured NSCs. After 48 h of transfection, the cells in the control group aggregated to form neurospheres and grew in suspension, while the cells in the shTtyh1 group protruded neurites and adhered to the plate. **(B)** Immunofluorescence staining of NSCs after transfection. After 72 h of transfection, the NSCs were collected and resuspended into single cell suspensions, plated on cover glass and incubated for 24 h, and then subjected to immunofluorescence staining. Compared with the control group, the proportions of Sox2^+^ cells **(left)** and EGFR^+^ cells **(middle) **in the shTtyh1 group were significantly reduced, whereas DCX^+^ cells **(right)** were not significantly changed. **(C)** Statistics of the percentages of immunopositive cells in B. **(D)** Immunofluorescence staining of differentiated neural cell markers. Compared with the control group, the proportions of GFAP^+^ and O4^+^ cells were increased, whereas the proportion of Map2^+^ cells were decreased. **(E)** Statistics of the percentages of immunopositive cells in D. Scale bar = 50 μm in A, Scale bar = 200 μm in B, D. *n* = 5 for all experiments. Data are expressed as mean ± SEM. Statistical significance was calculated using an unpaired, two-tailed Student’s *t*-test. (∗∗∗) *p* < 0.001, (∗∗) *p* < 0.01.

To further study the function of Ttyh1 in NSCs, we cultured primary NSCs from E14.5 mouse brains, and transfected NSCs with lentivirus expressing shTtyh1. The result showed that NSCs in the control group formed neurospheres in suspension 48 h after transfection, but NSCs in the shTtyh1 group grew adherently and protruded neurites (Figure 3A). Transfected NSCs were collected and dissociated into single cell suspension 72 h after transfection, and plated onto the cover glass coated with poly-L-lysine. After 24 h of culture, the control and shTtyh1-transfected NSCs were analyzed by immunofluorescence. The results showed that compared with the control, the proportions of Sox2^+^ cells and EGFR^+^ cells decreased significantly in the shTtyh1-transfected group (Figures 3B,C, *P* = 0.0033, *P* = 0.0017, respectively). Next, we examined the effect of Ttyh1 knockdown on NSC differentiation. Staining of neural differentiation markers showed that after Ttyh1 knockdown, the proportions of GFAP^+^ astrocytes and O4^+^ oligodendrocytes increased significantly (Figures 3D,E, *P* = 0.0009, *P* = 0.0002, respectively), and the proportion of Map2^+^ cells decreased (Figures 3D,E, *P* = 0.0026). Therefore, Ttyh1 knockdown *in vitro* damaged NSC stemness and promote their differentiations.

### Loss of Ttyh1 Leads to Activated Neurogenesis in Adult Mice

To further observe the role of Ttyh1 on NSCs *in vivo*, we knocked out exon 4 of the Ttyh1 gene through CRISPR/Cas9 system to construct Ttyh1 KO mice (Wu et al., 2019). We found that the viability and gross brain morphology of wild type control and Ttyh1 KO mice were comparable (Supplementary Figure S2). In accordance, no obvious defects were observed in NSCs on middle embryonic stage (Supplementary Figure S3). Therefore, we focused on NSCs in adult Ttyh1 KO mice aged 6–8 weeks.

**FIGURE 4:**
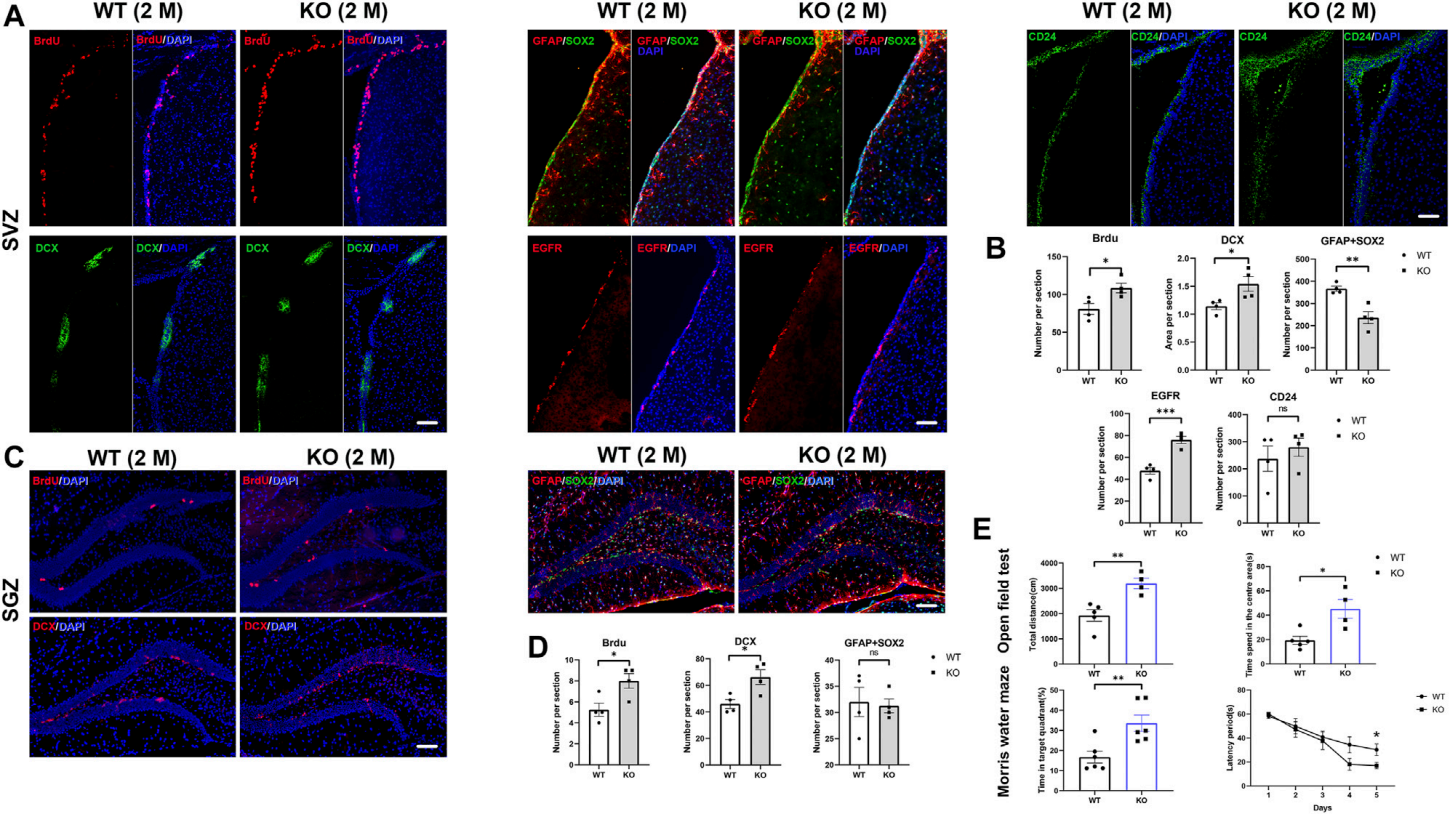
Loss of Ttyh1 leads to enhanced neurogenesis in adult mice. **(A,B)** Immunolabeling of cell-type-specific markers and quantification of immunopositive cells in the adult SVZ. The BrdU retaining cells, Sox2^+^GFAP^+^ NSCs, EGFR^+^ TAPs, DCX^+^ neuroblasts and CD24 ependymal cells were compared **(A)** and quantified **(B)** in SVZ between control and Ttyh1 KO mice at 2-months old (2 M). **(C,D)** Immunolabeling of cell-type-specific markers and quantification of immunopositive cells in the adult SGZ. The BrdU retaining cells, Sox2^+^GFAP^+^ NSCs, and DCX^+^ neuroblasts were compared **(C)** and quantified **(D)** in SGZ between control and Ttyh1 KO mice at 2 M. **(E)** Open field and Morris water maze tests between control and Ttyh1 knockout mice. Scale bar = 100 μm in A, C. *n* ≥ 4 for all experiments. Data are expressed as mean ± SEM. Statistical significance was calculated using an unpaired, two-tailed Student’s *t*-test. (∗∗∗) *p* < 0.001, (∗∗) *p* < 0.01, (∗) *p* < 0.05.

In order to label proliferating cells, BrdU incorporation assay was performed in 6–8 weeks Ttyh1 KO and control mice, followed by immunofluorescence staining using BrdU antibody. The results showed that the numbers of BrdU^+^ cells in SVZ and SGZ increased significantly in Ttyh1 KO mice compared with wild-type littermates (Figures 4A–D. *P* = 0.0281 in SVZ, *P* = 0.0271 in SGZ), indicating that NSC proliferation was enhanced in adult Ttyh1 KO mice. Then we estimated DCX^+^ neuroblasts by immunofluorescence staining. Consistently, the results showed that after Ttyh1 ablation, there were much more DCX^+^ neuroblasts in both SVZ and SGZ (Figures 4A–D. *P* = 0.0325 in SVZ, *P* = 0.0204 in SGZ), indicating that NSC differentiation was also enhanced. Altogether, these results revealed that Ttyh1 ablation leads to enhanced NSC proliferation and neurogenesis in adults.

Next, we estimated the number of type-B NSCs by double labeling of GFAP and Sox2, and observed that the double positive cells in the SVZ of adult Ttyh1 KO mice decreased compared with the wild type (Figures 4A–D. *P* = 0.0044). At the same time, the number of EGFR^+^ cells was higher than that of wild type (Figures 4A–D. *P* = 0.0007), suggesting that more NSCs differentiated from type-B NSCs into TAPs and DCX^+^ neuroblasts. However, immunofluorescence staining of hippocampus showed no obvious change of GFAP^+^Sox2^+^ cells in SGZ after Ttyh1 knockout (Figures 4C,D). We also tested the CD24^+^ ependymal cells between KO and control mice, and found that there were no significant changes (Figures 4A,B).

Activated neurogenesis in SGZ has been related to learning and memory, and anxiety (Gonçalves and Gage, 2016). To test behavior changes in Ttyh1-ablated mice, we carried out Morris water maze to evaluate the spatial learning and memory of mice. After 5 days of training, we carried out probe test and found that the latency of platform searching by Ttyh1 KO mice was significantly shorter than that of the wild type control (Figure 4E. *P* = 0.0362), and the time in the target area was obviously longer (Figure 4E. *P* = 0.0071). It has been shown that enhanced neurogenesis can make mice better adapting to new environments and reduce anxiety (Sahay et al., 2011; Gonçalves and Gage, 2016), so we further carried out the open field experiment. The results showed that the total distance and the time in the central area by Ttyh1 KO mice were longer than the wild type control (Figure 4E. *P* = 0.005, *P* = 0.0131), suggesting attenuated anxiety. These results suggested that Ttyh1 ablation improves learning and memory, and attenuates anxiety, consistent with enhanced neurogenesis in SGZ.

### Ttyh1 Prevents the Accelerated Exhaustion of NSC Pool in Aged Mice

**FIGURE 5:**
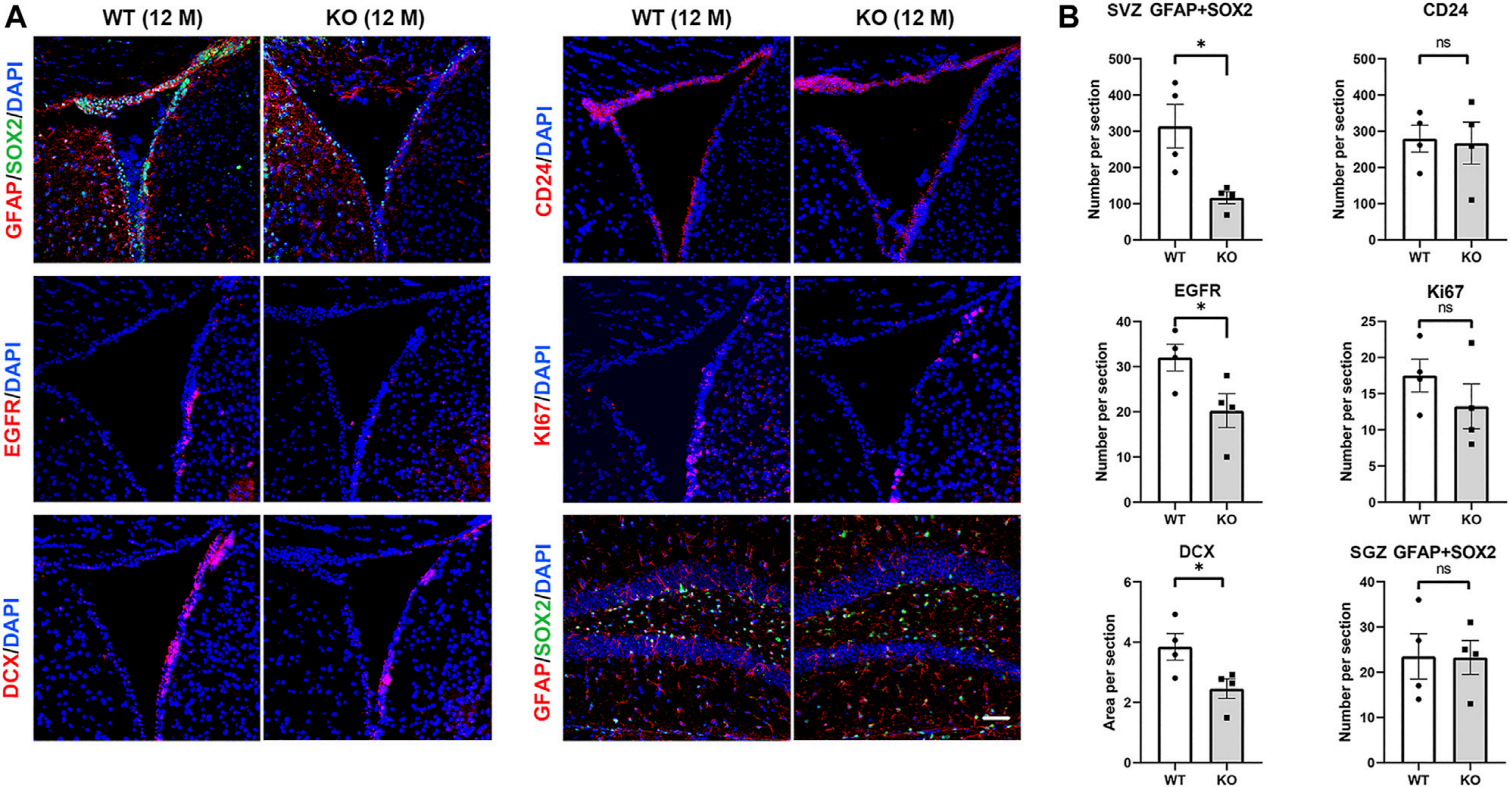
Ttyh1 prevents accelerated exhaustion of the elderly NSCs pool. **(A)** Immunofluorescence staining of brain sections of elder control and Ttyh1 KO mice at 12-months old (12 M). Sox2^+^GFAP^+^ NSCs, EGFR^+^ TAPs, DCX^+^ neuroblasts, Ki67 proliferating cells and CD24 ependymal cells were compared in neurogenic niche between control and Ttyh1 KO mice. **(B)** Statistics of immunofluorescence staining results in A. Scale bar = 50 μm in A. *n* = 4 for all experiments. Data are expressed as mean ± SEM. Statistical significance was calculated using an unpaired, two-tailed Student’s *t*-test. (∗) *p* < 0.05.

We then tested neurogenesis in aged (12-months) Ttyh1 KO mice. Immunofluorescence showed that the number of GFAP^+^Sox2^+^ cells in the SVZ was significantly lower in 12-months-old Ttyh1 KO mice than in the wild type control (Figures 5A,B, *P* = 0.0193). The number of EGFR^+^ cells and DCX^+^ cells in SVZ also decreased (Figures 5A,B, *P* = 0.0488, *P* = 0.0449). CD24^+^ ependymal cells were not affected in Ttyh1 knockout mice (Figures 5A,B). In addition, Ki67 staining showed no obvious change in cell proliferation in SVZ (Figures 5A,B). GFAP^+^Sox2^+^ NSCs in SGZ were comparable in Ttyh1 knockout mice compared with the control (Figures 5A,B). In summary, these results suggested that Ttyh1 can prevent NSCs from accelerated exhaustion in life span.

### Ttyh1 is Involved in the Regulation of Calcium Signaling Pathway

**FIGURE 6:**
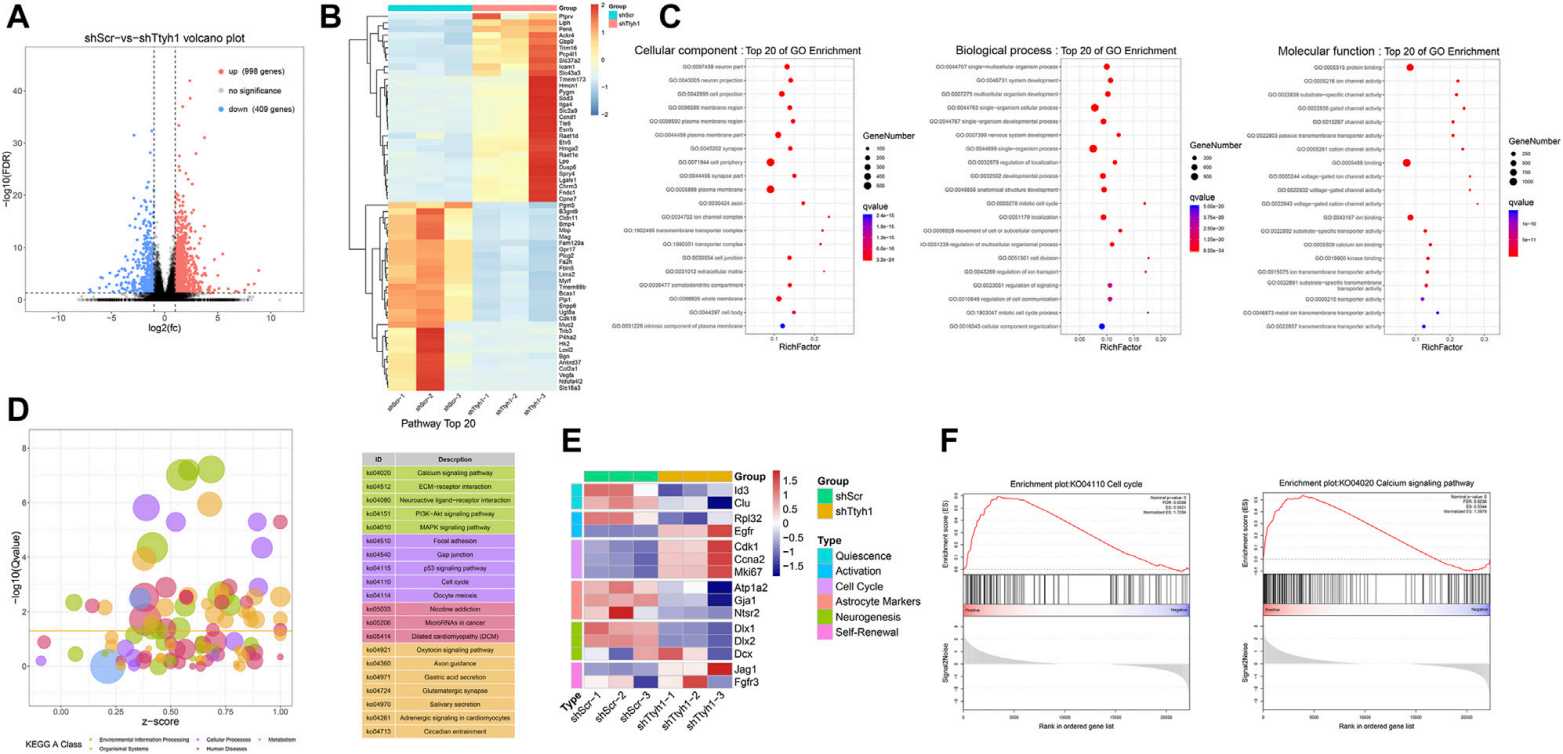
Transcriptome sequencing results after Ttyh1 knockdown indicate involvement of Calcium signaling and cell cycle genes. **(A)** Differential gene volcano plot. Red dots represent 998 up-regulated genes, and blue dots represent 409 down-regulated genes. We used false discovery rate (FDR) < 0.05 and fold change ≥2 as criteria to screen for genes with significant differences. **(B)** Heatmap of the top 30 genes with the most significant difference in up-regulation and down-regulation, respectively. Among them, cell cycle gene Ccnd1 (Cyclin D1) was significantly up-regulated in the Ttyh1 knockdown group. **(C)** Gene Ontology (GO) analysis. Colors represent q values on a log scale (with red corresponding to the most highly significant). Node size represents the number of genes in a category. Top 20 items of q value are listed.**(D)** KEGG pathway analysis. The vertical coordinate is −log10 (q value), and the horizontal coordinate is Z-score value (the proportion of the difference between the number of up-regulated genes and the number of down-regulated genes in the total differential genes), and the yellow line represents the threshold of *p* = 0.05. On the right is a list of signal pathways with the top 20 *p*-values, with Calcium signaling mostly significant. Different colors represent different categories. Green represents environmental information processing, purple represents cellular processes, red represents human diseases, yellow represents organismal systems, and blue represents metabolism. **(E)** Heatmap of stage-specific markers of NSCs between control and Ttyh1 knockdown groups. **(F)** GSEA (gene set enrichment analysis). After Ttyh1 knockdown, calcium signaling pathway and cell cycle related genes were interfered.

To explore the mechanism of Ttyh1 affecting NSC stemness and quiescence/activation transition, we knocked down Ttyh1 in cultured neurospheres from E14.5 embryos with shTtyh1 lentivirus, and performed RNA sequencing. The results showed that 998 genes were up-regulated and 409 genes were down-regulated in neurospheres transfected with shTtyh1 (Figure 6A). With false discovery rate (FDR) < 0.05 and fold change ≥2 as a standard, the top 30 most significantly increased or decreased genes were listed and displayed with a heatmap (Figure 6B). Ccnd1 (cyclin D1) was among the top 30 elevated genes (Figure 6B, *P* = 1.40E-06), suggesting that Ttyh1 knockdown leads to increased cyclin level, driving NSCs into cell cycle from a quiescent state.

Moreover, Ttyh1 knockdown significantly downregulated Id3 and Clu, markers of qNSCs (Figure 6E, *P* = 8.55E-05, *P* = 1.79E-04), consistent with the loss of quiescence in Ttyh1 deficiency NSCs. Ttyh1 knockdown resulted in significantly up-regulated expression of EGFR (Figure 6E), a typical signature of the transition from qNSCs to aNSCs (Dulken et al., 2017). The decrease of astrocyte markers Atp1a2, Gja1, and Ntsr2 (Figure 6E, *P* = 0.0436, *P* = 1.43E-04, *P* = 1.98E-04) suggests that Ttyh1 knockdown NSCs are in the transition from the middle stage to the late stage of aNSCs (Dulken et al., 2017). These results indicate that upon Ttyh1 knockdown, NSCs transit from quiescent into an activated state with cell cycle entry.

Gene Ontology (GO) analysis suggests that the differentially expressed genes are mainly involved in neuron projection, ion channel complexes, and the structure of the plasma membrane, and are involved in the development of the nervous system, the mitotic cell cycle, and the regulation of cell communication (Figure 6C), consistent with previous studies (Kumada et al., 2010; Kim et al., 2018; Wu et al., 2019). KEGG pathway analysis showed that after Ttyh1 knockdown, many signal pathways such as calcium ion signal pathway, p53 signal pathway, PI3K-Akt signal pathway, and cell cycle were activated, among which calcium signal pathway was the most significant one (Figure 6D). Genes including calcium channel proteins on the cell membrane (*CaV2*, *CaV3*, *ROC*) and ER (*STIM1*, *RYR*), and genes downstream of the calcium pathway (*CALM*, *CaMKII*), were up-regulated (data not shown). GSEA analysis confirmed these findings (Figure 6F). In summary, Ttyh1 knockdown upregulates cell cycle-related genes, accompanied by the alteration of the calcium signaling pathway-related genes.

### NFATc3 is a Key Transcription Factor in Regulating NSC Stemness by Ttyh1

**FIGURE 7:**
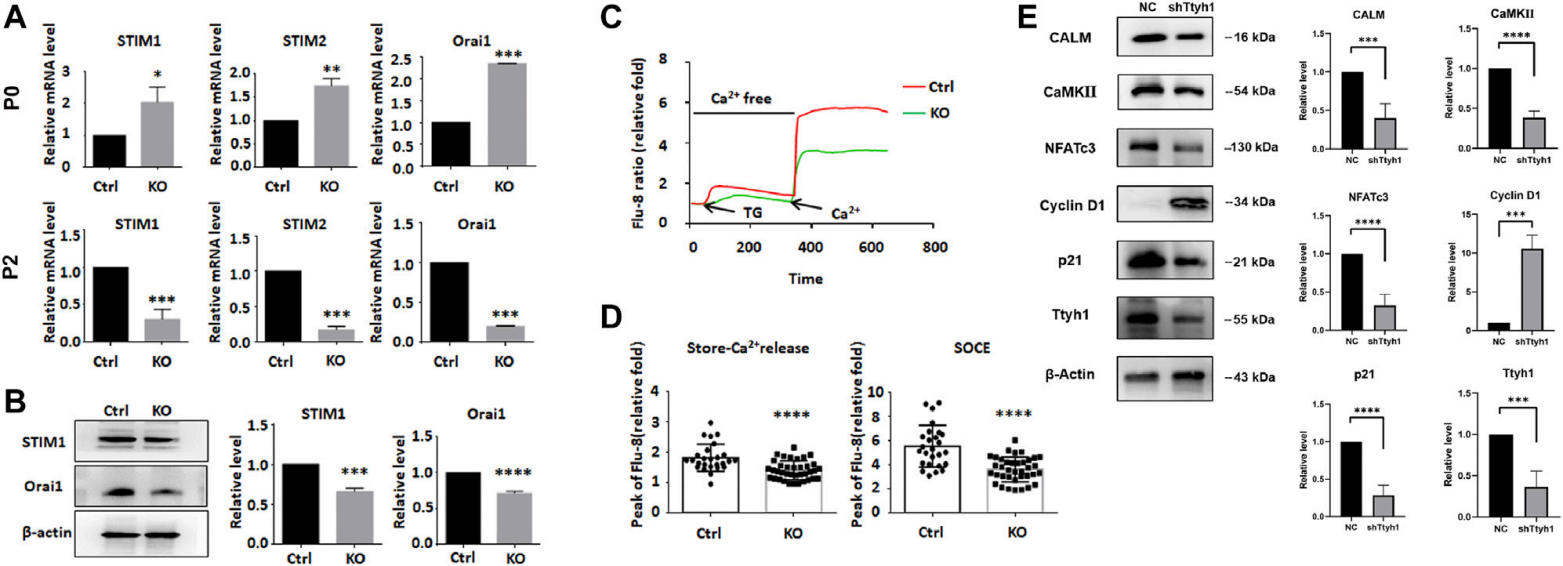
Ttyh1 is involved in the regulation of calcium signaling in NSCs. **(A)** RT-qPCR analysis showed the expression levels of SOCE regulatory molecules between control and Ttyh1 KO neurospheres at primary culture (P0) and Passage 2 (P2). The RNA expressions of STIM1, STIM2, and Orai1 increased at P0, and decreased at P2 generation. **(B)** Western blotting analysis showed the protein levels of STIM1 and Orai1 between control and Ttyh1 KO neurospheres in P2 generation. **(C,D)** Intracellular calcium measurement between control and Ttyh1 KO NSCs. We used Flu8 to label the calcium ions in NSCs, then add 10 μM Thapsigargin (TG) to induce store-Ca^2+^ release from the endoplasmic reticulum, and finally add 2 μM calcium chloride solution to detect the change of store-operated Ca^2+^ entry (SOCE). The results showed that both store-Ca^2+^ release and SOCE reduced significantly in P2 generation of Ttyh1 knockout NSCs. **(E)** Western blotting analysis of downstream molecules of Calcium signaling pathway. Representative Western blots **(left)** and quantification of bands intensities **(right)** are present. After Ttyh1 was knockdown by shRNA containing lentivirus, the protein levels of CALM, CaMKII, NFATc3, and p21 were decreased, and that of Cyclin D1 increased significantly. *n* ≥ 4 for all experiments. Data are expressed as mean ± SEM. Statistical significance was calculated using an unpaired, two-tailed Student’s *t*-test. (∗) (∗∗∗∗) *p* < 0.0001, (∗∗∗) *p* < 0.001, (∗∗) *p* < 0.01, (∗) *p* < 0.05.

We examined the expression of the key components involved in extracellular calcium entry and ER calcium release, including STIM1, STIM2, and Orai1 (Clapham 2007; Son et al., 2020). Consistent with the transient increase and subsequent decrease of proliferation and stemness of Ttyh1 knockout neurospheres from E14.5 embryos (Wu et al., 2019), the mRNA levels of STIM1, STIM2 and Orai1 increased in the primary cultured (Passage 0, P0) knockout group (Figure 7A, *P* = 0.0191, *P* = 0.0013, *p* < 0.001), but decreased significantly in the Passage 2 (P2) generation (Figure 7A, *p* < 0.001, *p* < 0.001, *p* < 0.001) and remained stable thereafter. Western blotting showed that STIM1 and Orai1 were significantly decreased after Ttyh1 knockout (Figure 7B, *P* = 0.0002, *p* < 0.0001). We then detected changes in the intracellular calcium concentration, and the results showed that both the calcium release from the intracellular calcium store (Figures 7C,D, *p* < 0.0001) and the store-operated calcium entry (SOCE) (Figures 7C,D, *p* < 0.0001) were significantly reduced, leading to a decrease in the intracellular calcium level. Then we measured the expression of CALM, an important downstream molecule of calcium (Burgoyne 2007), in neurospheres with Ttyh1 knockdown, and found that the expression of CALM decreased significantly (Figure 7E, *P* = 0.0007). The expression of CaMKII, a Ca^2+^/CALM-dependent protein kinase, also showed a significant decrease (Figure 7E, *p* < 0.001). Moreover, the expression of cyclin D1 was significantly up-regulated (Figure 7E, *P* = 0.0007), while p21 was significantly reduced (Figure 7E, *p* < 0.0001), after Ttyh1 knockdown. These results suggested that cytosolic Ca^2+^ level decreased and calcium pathway-related proteins were down-regulated after Ttyh1 ablation, affecting the expression of cyclin and regulating cell cycle in NSCs.

**FIGURE 8:**
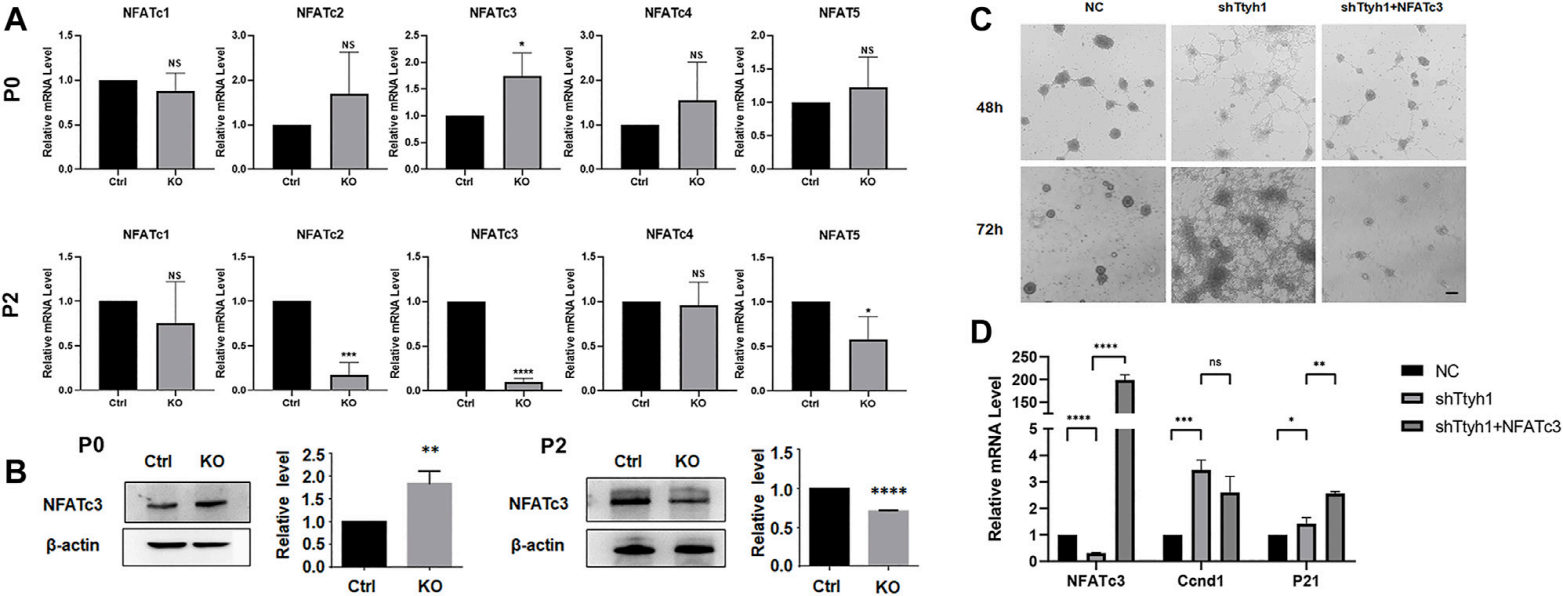
NFATc3 is a key transcription factor in the regulation of cell cycle by Ttyh1. **(A)** RT-qPCR analysis showed the expression levels of five NFAT family members between control and Ttyh1 KO neurospheres at primary culture (P0) and Passage 2 (P2). The RNA expression of NFATc3 increased at P0 and decreased significantly at P2. **(B)** Western blotting analysis showed the protein level of NFATc3 was also increased at P0 but decreased at P2. **(C)** Bright field microscopy showed the morphological disparity among NSCs transfected with negative control shRNA (NC), shTtyh1, and shTtyh1+ NFATc3 (rescue group), respectively. After 48 or 72 h of transfection, the results showed that overexpression of NFATc3 partially rescued the phenotypic changes caused by knockdown of Ttyh1. **(D)** RT-qPCR analysis showed that when Ttyh1 was knockdown, Ccnd1 was significantly increased, and p21 was slightly increased too. When NFATc3 was overexpressed at the same time, p21 increased significantly, whereas Ccnd1 showed a down-regulated trend. Scale bar = 100 μm in C. *n* ≥ 4 for all experiments. Data are expressed as mean ± SEM. Statistical significance was calculated using an unpaired, two-tailed Student’s *t*-test. (∗∗∗∗) *p* < 0.0001, (∗∗∗) *p* < 0.001, (∗∗) *p* < 0.01, (∗) *p* < 0.05.

Calcium signaling pathway can affect the cell cycle through downstream NFAT family molecules (Clapham 2007), and the calcineurin/NFAT axis has been reported to regulate NSCs and neurogenesis (Quadrato et al., 2012; Quadrato et al., 2014; Artegiani et al., 2015; Moreno et al., 2015). We then tested the mRNA expression of *NFAT*s in P0 and P2 generations of neurospheres from Ttyh1 knockout mice (Figure 8A). NFATc3 showed up-regulation at P0 and down-regulation at P2 (Figure 8A, *P* = 0.0413, *p* < 0.0001), consistent with the expression of STIM1 and Orai1. Western blotting also proved that NFATc3 increased in P0 and then decreased in P2 generations of Ttyh1 deficient neurospheres (Figure 8B, *P* = 0.0062, *p* < 0.0001).

To verify that Ttyh1 regulates the stemness of NSCs through NFATc3, we conducted rescue experiments. Cultured NSCs were transfected with lentivirus expressing shTtyh1or shTtyh1 plus active NFATc3 (Li et al., 2011), and observed 48 and 72 h after the transfection (Figure 8C). The results showed that NSCs of the knockdown group began to grow adherently and formed cell processes 48 h after the transfection (Figure 8C). In the shTtyh1 and active NFATc3 co-transfection group, neurospheres maintained the same morphology as the control (Figure 8C). After 72 h of transfection, cells in the co-transfection group still formed small neurospheres without significant adherent growth or neurite protrusion. These results proved that overexpression of NFATc3 could partially rescue the phenotypic changes caused by knockdown of Ttyh1.

## Discussion

In this study, we determined that transmembrane protein Ttyh1 is mainly expressed in Ki67 negative qNSCs. Ttyh1 knockout leads to NSC activation and enhanced neurogenesis in adult mice, with behavioral consequences. Correspondingly, after overactivated neurogenesis in adulthood, aged Ttyh1 KO mice show the exhaustion of NSCs. The loss of quiescence, enhanced neurogenesis, and the exhaustion of NSCs in Ttyh1 KO mice is remarkably similar to the phenotypes observed after Notch inhibition in adult NSCs in previous report (Imayoshi et al., 2010). We have reported that the expression of Ttyh1 is regulated by Notch signaling in the previous work (Wu et al., 2019). Therefore, Ttyh1 is likely a putative Notch target gene in the brain, and the precise mechanism by which Notch signaling regulates Ttyh1 needs further study. On the other hand, although the conventional Ttyh1 KO mice are viable and grossly normal (Supplementary Figures S2, S3), the probable non-cell autonomous defects of NSCs indirectly affected by subtype phenotypes could not be excluded. Therefore, conditional and inducible Ttyh1 knockout models in NSCs are needed to further confirm its function in the future.

RNA-seq suggests that calcium signaling underlies the Ttyh1-regulated NSC quiescence/activation transition. The C terminal of Ttyh1 is enriched with aspartates that can bind Ca^2+^ (Kumada et al., 2010). Our further analysis has revealed that intracellular Ca^2+^ was decreased and the key regulators of SOCE were down-regulated under Ttyh1 deficiency. Recently, Gengatharan et al. reported that several microenvironmental signals converge on intracellular Ca^2+^ pathways to regulate NSC quiescence and activation (Gengatharan et al., 2021). Another research work on human NSCs also revealed that stretch-activated Piezo1 directs NSC lineage specification by eliciting Ca^2+^ influx, therefore transducing matrix mechanical cues to intracellular signaling pathways (Pathak et al., 2014). Together with our findings, these results suggest Ca^2+^ as a possible mechanism for integrating and decoding multiple extrinsic signals governing NSC quiescence/activation transition and specification. On the other hand, the neurospheres in our experiment were obtained from embryonic mouse brains, and whether the same mechanism exists in adult NSCs needs further confirmation.

Our data suggested that Ttyh1 knockdown appears to increase the mRNA level of *CALM* and *CaMKII* as shown by RNA-seq, but reduce their protein level shown by Western blotting. Similar phenomenon was also reported by Kjell et al. (2020). The possible reason could be that the expression of the calcium pathway-related genes increased briefly and then decreased continuously in our system. Moreover, unknown differential regulations in transcription and translation might cause differences in mRNA and protein levels.

Our rescue experiments have confirmed that NFATc3 plays an important role in maintaining the stemness of NSCs downstream to Ttyh1. Previous studies have confirmed that NFATc1 and NFATc2 can regulate cell cycle progression through Cyclin D1 and p21 (Mognol et al., 2016). We proved that Ttyh1 could regulate cyclin D1 and p21 expression through NFATc3. After Ttyh1 was knocked down, the expression of cyclin D1 was significantly increased. And the expression of p21 was significantly increased after NFATc3 overexpression. This suggests that the relative amount of cyclin D1 and p21 determines cell cycle entry in NSCs. Chen et al. have reported that Dyrk1a can regulate the expressional balance of cyclin D1 and p21 to determine cell cycle entry in their studies of Down’s syndrome (Chen et al., 2013), and they concluded a p21-cyclin D1 signaling map, directing each cell to either proliferate or to follow cell cycle exit characterized by high or low cyclin D1 and p21 levels. In our study, NFATc3 seems to play a similar role in cell cycle regulation of NSCs by modulating cyclin D1 and p21. This integrative regulation mode provides a subtle and flexible mechanism for cells to exit and re-enter cell cycle, so that NSCs have different developmental and physiological states within neurogenic niches. In summary, this regulatory model is of great significance for precisely regulating the quiescence/activation transition of NSCs and provide a utilization target of the repair potential of NSCs.

Finally, Ttyh1 is also expressed in differentiated neural cell types especially astrocyte. The gene expression of astrocytes is very close to that of qNSCs. They share many molecular markers, and Ttyh1 is one of them. This is consistent with that astrocytes retain certain characteristics and functions of NSCs (Kriegstein and Alvarez-Buylla 2009). Whether Ttyh1 participates in astrocytes-mediated repair of the nervous system damage is worthy of further study.

## Data Availability

The original contributions presented in the study are publicly available. This data can be found here: National Center for Biotechnology Information (NCBI) BioProject database under accession number PRJNA771085.
