# Supplementary material for: Transmembrane Protein Ttyh1 Maintains the Quiescence of Neural Stem Cells Through Ca2+/NFATc3 Signaling
Source: Front Cell Dev Biol. 2021 Nov 16;9:779373. doi: 10.3389/fcell.2021.779373 (PMC8635056; doi:10.3389/fcell.2021.779373)
Supplement: Supplementary file 1 [file DataSheet1.docx]

Supplemental Information

Supplemental figure 1:


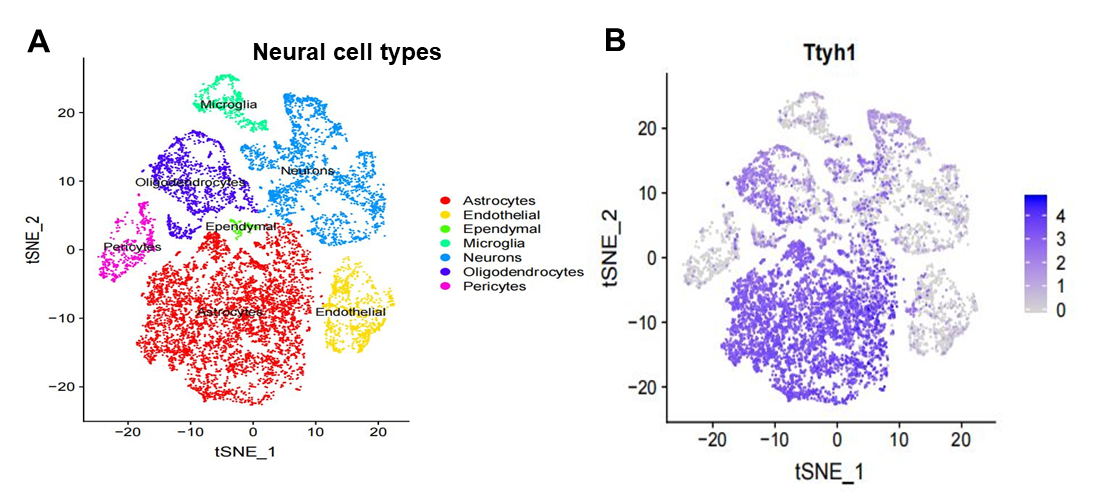


Sup Figure 1: The expression of Ttyh1 in mature neural cell types. A. Single cell cluster analysis showed the classification of mature neural cell types. B. Single-cell cluster analysis showed the distribution of Ttyh1 in adult neural cell types. Purple represents Ttyh1^+^ cells, which mainly were astrocytes and oligodendrocytes.

Supplemental figure 2:


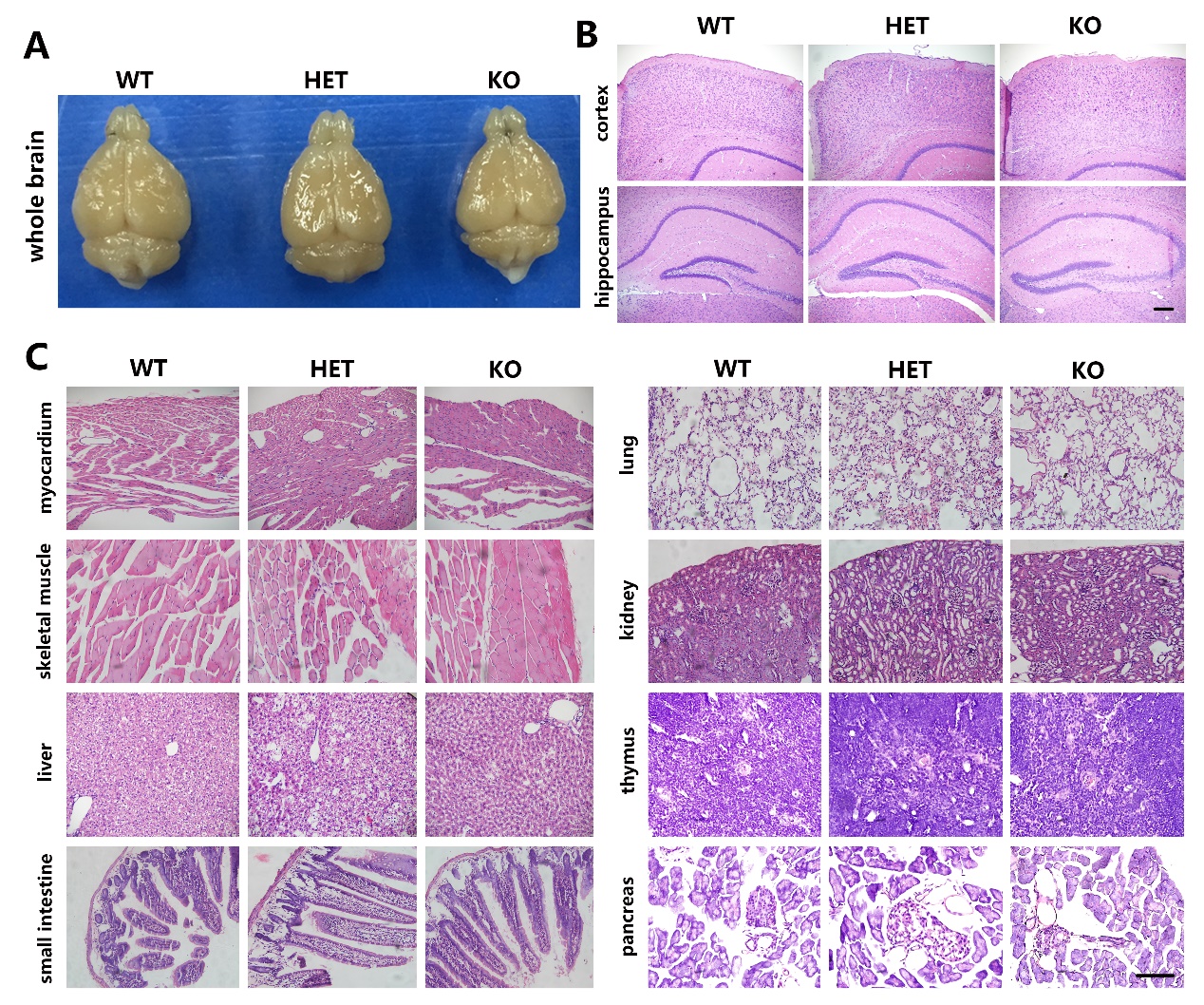


Sup Figure 2: The brain morphology and H&E staining of organ sections from wild type (WT), Ttyh1+/- (HET) and Ttyh1-/- (KO) mice were comparable. A. brain morphology. B. H&E staining of cortex and hippocampus. C. H&E staining of various organ sections. Scale bar = 100 μm in B and C.

Supplemental figure 3:


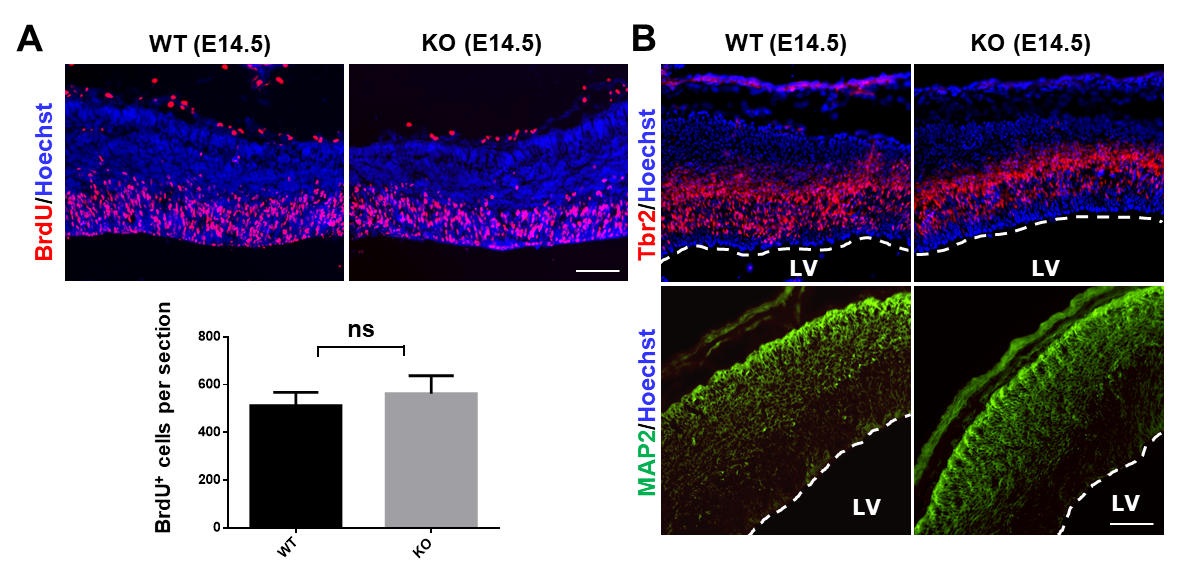


Sup Figure 3: The proliferation and differentiation of embryonic NSCs are grossly normal upon Ttyh1 ablation. A. BrdU labeling and statistic result of E14.5 brain sections showed no obvious change of NSC proliferation in Ttyh1 knockout embryos compared with wild type littermates. B. Tbr2 and MAP2 immunostainings showed that the differentiation of NSCs was comparable between Ttyh1 knockout and wild type embryonic brains. Scale bar = 100 μm in A and B. LV, lateral ventricle.
